# Identification of the Brassinazole-Resistant (*BZR*) Gene Family in Wheat (*Triticum aestivum* L.) and the Molecular Cloning and Functional Characterization of *TaBZR2.1*

**DOI:** 10.3390/ijms252312545

**Published:** 2024-11-22

**Authors:** Yan Zhang, Jingzi Qin, Jinna Hou, Congcong Liu, Shenghui Geng, Maomao Qin, Wenxu Li, Ziju Dai, Zhengqing Wu, Zhensheng Lei, Zhengfu Zhou

**Affiliations:** 1 Henan Institute of Crop Molecular Breeding, Henan Academy of Agricultural Sciences, Zhengzhou 450002, China; zhangyan123@hnagri.org.cn (Y.Z.); qinjingzi@hnagri.org.cn (J.Q.); houjinna@hnagri.org.cn (J.H.); liucongcong@hnagri.org.cn (C.L.); gengshenghui@hnagri.org.cn (S.G.); qinmaomao@hnagri.org.cn (M.Q.); liwenxu@hnagri.org.cn (W.L.); daiziju@hnagri.org.cn (Z.D.); wuzhengqing@hnagri.org.cn (Z.W.); 2 National Key Laboratory of Wheat and Maize Crop Science, College of Agronomy, Henan Agricultural University, Zhengzhou 450002, China; 3 The Shennong Laboratory, Zhengzhou 450002, China

**Keywords:** wheat, transcription factor, *BZR*, expression profiles

## Abstract

Brassinazole-resistant (BZR) transcription factors are important transcription factors in Brassinosteroid (BR)-responsive gene expression. However, limited knowledge exists regarding the *BZR* genes in wheat and a limited number of *BZR* family genes have been previously reported in wheat. In this study, the synteny analyses of the *TaBZR* genes suggested that gene duplication events have played an essential role in the *TaBZR* family during evolution. The results of RT-qPCR and transcriptome data analyses exhibited remarkable expression patterns in the *BZR* genes in different tissues and under different treatments. The yeast two-hybrid (Y2H) screen result showed that the TaBZR2.1 protein interacts with Argonaute 4 (AGO4). Taken together, our results not only provide us a basis for understanding the molecular characteristics and expression patterns of the *TaBZR* family genes but also offered the functional characterization of *TaBZR2.1* in wheat.

## 1. Introduction

Brassinosteroid (BR) is a kind of plant-specific steroidal hormone that plays a crucial role in many plant biological process, such as regulating plant growth, development, and the stress response [1]. It is reported that mutant plants with defects in BR biosynthesis or BR signal components display characteristic phenotypes such as dwarfism [2], constitutive photomorphogenesis in darkness [3], altered fertility [4], changes in seed size and number [5,6,7], delayed flowering, and defects in xylem differentiation [8]. Unlike animal steroid hormones, plant BRs are perceived by receptors that are located on the cell membrane [9]. Upon receiving the BRs’ signal, the cell membrane receptor kinase BRASSINOSTEROID INSENSITIVE1 (BRI1) is activated and can phosphorylate a negative regulator, BRI1 kinase inhibitor 1 (BKI1), inducing its dissociation from the cell membrane [10]. Many plant transcription factors are located downstream of BR signaling events, including key factors such as BRASSINAZOLE-RESISTANT 1 (BZR) [11]. Acting as positive regulators in BR signaling, *Arabidopsis* BES1 and BZR factors are two BES/BZR members of the *BZR* family [12].

BZR acts as an important regulator in maintaining BR homeostasis for plant growth and development [13]. It mediates BR-induced gene expression and suppresses BR biosynthesis through feedback mechanisms [11]. BZR transcription factors induce the BR-induced genes [11,14,15,16]. In *Arabidopsis*, BZR can be phosphorylated by activating BIN2 and interacting with 14-3-3s to regulate BR signaling, which retained BES1/BZR in the cytosol to prevent BR signaling [17,18]. Dephosphorylation of BZR by PP2A allows it to transfer to the nucleus to regulate BR-induced gene expression [19]. BZR also mediates the ovule and seed number in *Arabidopsis* [5]. It has been reported that BR-activated BZR directly regulates the genes involved in seed size control such as ZmBES1/BZR-5, which positively regulates kernel size by inhibiting *AP2*/*EREBP* gene transcription [6,7]. Overexpression of *ZmBZR* transgenic *Arabidopsis* results in large organs and seeds [20].

Studies have firmly established that plant development and growth are synergistically regulated by environmental and hormonal signals, with light and BR serving as the two central stimuli regulating plant morphogenesis. It is reported that certain photoreceptors mediate brassinosteroid signaling by interacting with BZR. For instance, by interacting with the photoreceptor phytochrome B (phyB), BZR can repress BR signaling in *Arabidopsis* [21]; by interacting with CRY1, BZR and BIN2 can repress BR signaling [22]; and the interaction of PIF4 and BZR controls a hub of transcription networks, integrating various endogenous and environmental signals [23]. The interaction of BZR and the HY5 mediates the BR and light signaling pathways to regulate the cotyledon opening in seedling photomorphogenesis in *Arabidopsis* [24]. Additionally, BZR also plays a vital role in the response to nitrogen starvation in tomatoes [25].

Furthermore, certain abiotic stresses can induce the expression of *BZRs*, conferring tolerance to stresses. For example, a *TaBZR* gene could be significantly upregulated by salt treatment in wheat, improving salt tolerance through activating the genes involved in ROS scavenging and ABA biosynthesis [26]. Tomato BZR can regulate heat stress responses by ROS signaling [27]. Ectopic expression of a maize *BZR* gene in *Arabidopsis* confers tolerance to osmotic stress [28]. Through CBF-dependent pathways, BZR positively modulates plant freezing tolerance in *Arabidopsis* [29]. Ectopic expression of the maize *ZmBZR1* gene in rice and *Arabidopsis* negatively regulated drought tolerance [30].

The BZR transcription factors have been identified in numerous species [31,32,33]. Wheat is a globally significant cereal crop globally, providing approximately 20% of the total calories consumed by humans [34]. However, compared to other crop species, our understanding of the BZR transcription factors in wheat remains limited. We do not know whether the wheat *BZR* gene also responds to plant hormones, participates in the stress response, or has a role in seed development. This comparative analysis on gene/protein structure, evolution history, and expression pattern will help to reveal the molecular function of *BZR* family genes and provide gene resources for wheat breeding. Therefore, this study characterized 20 members of the wheat *BZR* gene family and assessed their expression profiles. Additionally, we further characterized the interacting protein of TaBZR2.1 using Y2H assays and performed subcellular localization assays. The findings from this investigation may facilitate further investigation of the molecular functions of the *BZR* family genes in wheat.

## 2. Results

### 2.1. Identification of TaBZR Genes in Wheat

**Figure 1:**
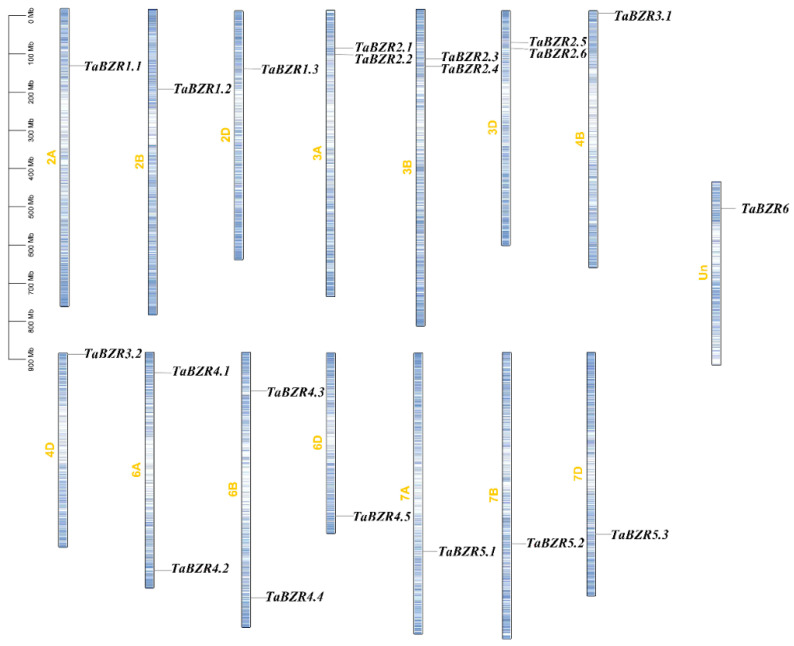
The chromosome distribution of the *TaBZR* genes.

The synteny analyses and chromosomal localization of *TaBZR* genes showed that 19 out of the 20 *TaBZR* genes were distributed in 14 of the chromosomes (Figure 1), and the 1 remaining *TaBZR* gene was mapped onto an unattributed scaffold. With the exception of the *BZR* genes on 4B and 4D, the distribution and number of *TaBZR* genes from the A/B/D subgenomes were similar. Among them, *TaBZR4.4* had four transcripts, *TaBZR6* had three transcripts, and *TaBZR4.1* had two transcripts. The CDS and polypeptide length, MW, transcript number, pI, and the predicted subcellular localization were analyzed and shown in Table S1.

### 2.2. The Exon–Intron Structure of TaBZR Genes

**Figure 2:**
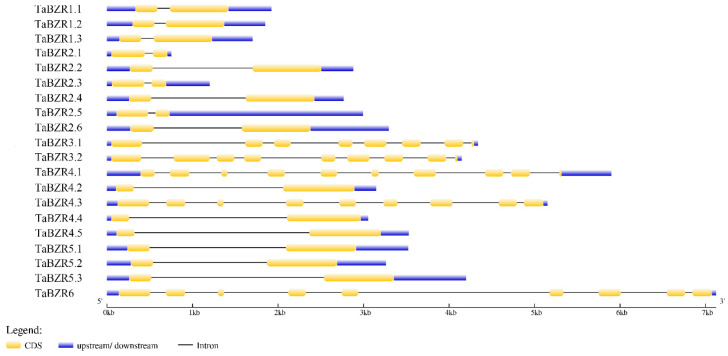
The genomic structure of *TaBZR* genes. The blue boxes in the schematic diagram represent the upstream/downstream sequences; the exon sequences and the introns are represented by yellow boxes and black lines, respectively.

The gene structures of 20 wheat *BZRs* were investigated in this study (Figure 2). The results revealed that the intron and exon numbers of the *TaBZRs* were different from *TaBZR1.1* to *TaBZR6*. The numbers of exons differed from two to ten, and most of the *TaBZR* genes contained the minimum exon number, i.e., two. Meanwhile, *TaBZR4.1* had ten, and *TaBZR3.1*/*3.2*/*4.3*/*6* contained eight or nine exons. It has been reported that the *TaBZR* genes in tomatoes, Chinese cabbage, and apples do not contain the upstream and downstream structures [35]. Meanwhile, all of the *TaBZR* genes in wheat contained these structures, and all of them had exons and introns.

### 2.3. Analysis of Three-Dimensional Modeling

**Figure 3:**
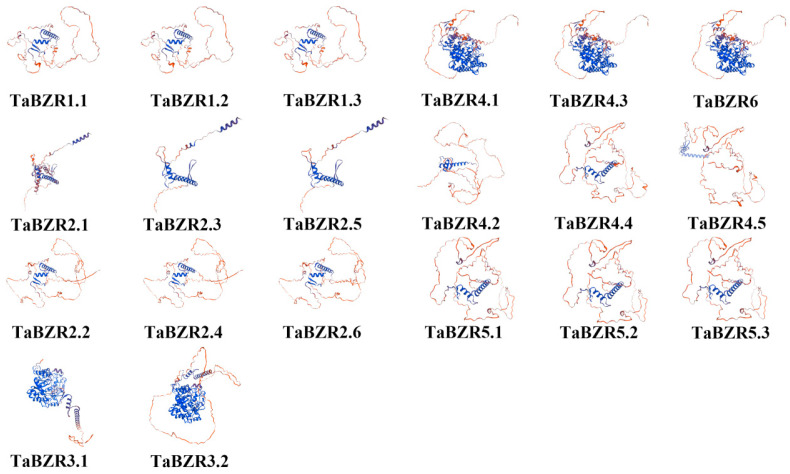
The tertiary structure prediction of the BZR protein in wheat. The tertiary structures of the TaBZR proteins were generated using the SWISS-MODEL. Twenty BZR proteins were modeled based on GMQE.

To further investigate the protein structural effects of the BZRs in wheat, a three-dimensional (3D) model of BZR proteins was constructed on the SWISS-MODEL website, and the optimal model was selected according to Global Model Quality Estimation (GMQE) (Figure 3). The results showed that their structural integrity was preserved throughout their evolution, which is crucial for their function. A phylogenetic analysis of wheat BZR genes was conducted in a previous study, and our results strongly support the reliability of their phylogenetic analysis of the BZR genes [36].

### 2.4. Synteny Analyses of TaBZR Genes

**Figure 4:**
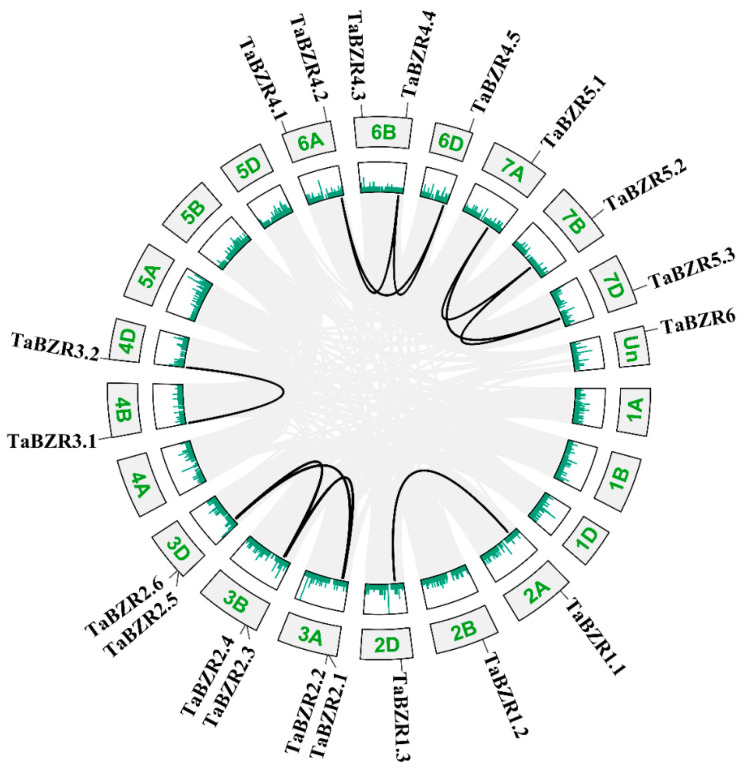
The interchromosomal relationships of the *TaBZR* gene family in wheat. In the middle of the figure, the black lines indicate the *TaBZR* gene pairs, and the gray lines represent all of the synteny blocks in the wheat genome. The green bars on each chromosome represent gene density. The number of each chromosome is indicated in green.

Gene duplication events are known as an important source of gene complexity diversity [37]. Synteny analyses of the *TaBZR* genes were performed to better understand *TaBZR* gene family gene duplication and expansion events in wheat (Figure 4). Gene duplication acts a major factor leading to expansion of the gene family. Current hexaploid wheat was produced as a result of two natural hybridization events between three diploid species. In theory, every wheat gene often has three homologous genes, and they are caused by polyploidization [38]. Figure 4 showed that the *TaBZRs* were distributed on the 14 wheat chromosomes. Fourteen *TaBZR* genes (*TaBZR-1.1*/*1.2*/*1.3*, *TaBZR2.1*/*TaBZR2.2*/*TaBZR2.3*, *TaBZR3.1*/*TaBZR-3.2*, *TaBZR4.2*/*TaBZR4.4*/*TaBZR4.5*, and *TaBZR5.1*/*TaBZR5.2*/*TaBZR5.3*) were clustered into eleven tandem duplication event regions. These results proved that gene duplication events are an important driving force during *TaBZR* gene family evolution.

### 2.5. Cis-Regulatory Elements in the Promoter Region of TaBZRs

**Figure 5:**
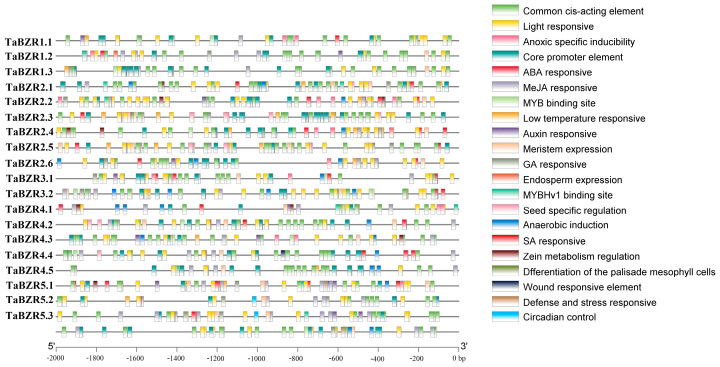
The cis-elements in the promoter regions of the *TaBZR* genes.

The promoter region of many of the genes contained many cis-acting elements. The cis-elements in the promoter regions of the *TaBZR* genes were identified. To understand the stress-responsive regulatory role of the *BZR* genes in wheat, promoter cis-acting elements of *TaBZR* genes were further analyzed (Figure 5). There were many cis-elements in the 20 *TaBZR* genes’ promoter regions, such as anoxic-specific inducibility elements, anaerobic induction elements, low-temperature-responsive elements, and some plant-hormone-responsive elements, such as MeJA, ABA, GA, and SA response elements. Some of the *TaBZR* gene promoters contain elements associated with the seed-specific regulation element, light-responsive element, and meristem expression. Additionally, MYB TF binding sites involved in light and drought are also found in the promoter regions.

### 2.6. Conserved Motifs of TaBZRs

**Figure 6:**
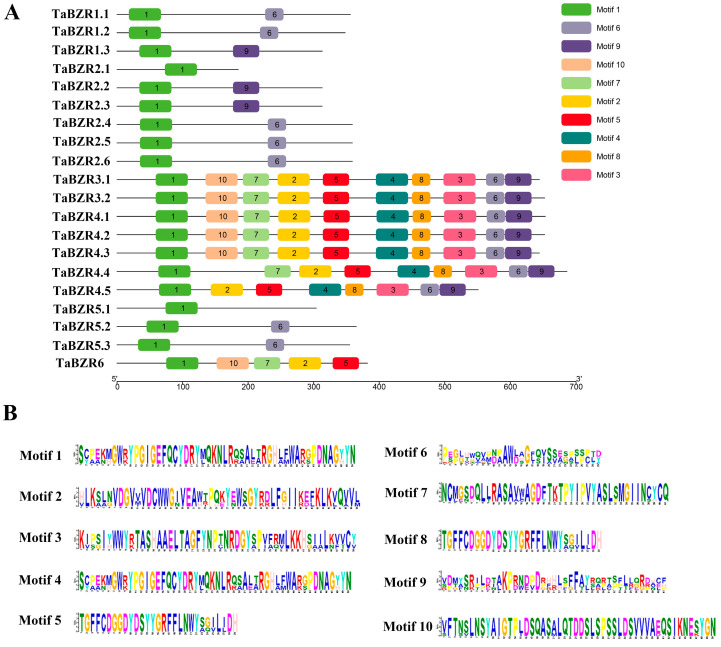
Distribution of the conserved motifs of the TaBZRs. (**A**). Conserved motif analysis of TaBZR was performed in this study. The different colored boxes numbered 1–10 indicate different motifs. The annotations of the motifs are listed on the right. (**B**). The conserved amino acid sequences in each motif.

The protein motifs of the TaBZR protein were analyzed utilizing MEME online tools (Figure 6). We found that motif 1 was conserved in most of the TaBZR transcripts, suggesting that this region was important for BZR protein functions. Some of the motifs were found only in a few of the TaBZR subfamilies. For example, motifs 2, 3, 4, 5, 7, 8, and 10 were specific to the subfamily group II. The results of the conserved motif analysis of TaBZR were generally consistent with those of the TaBZR phylogenetic analysis.

### 2.7. Expression Profiles of Wheat BZR Genes

**Figure 7:**
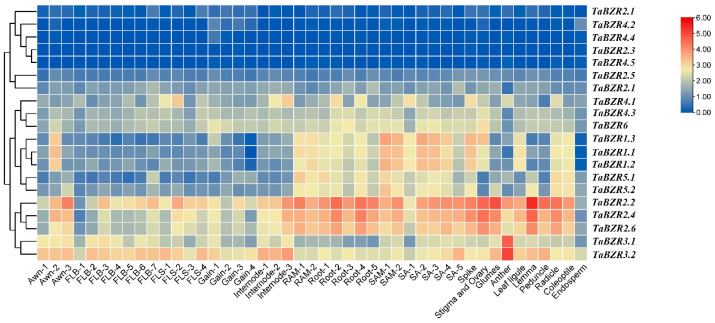
The wheat *BZR* gene expression profiles. Hierarchical clustering of the wheat *BZR* gene expression profiles in 45 of the samples, including different tissues and development stages. The numbers in the schematic diagram represent the development stages of the same tissue.

Published data were downloaded and analyzed in this study to gain insight into the spatial and temporal expression profiles of wheat *BZR* genes, and the expression patterns of all 20 *BZR* genes were derived from different development stages of the wheat tissues that were investigated (Figure 7). The result revealed that some of the *TaBZR* genes showed preferential expression in some detected tissues. For example, two genes in anther (*TaBZR3.1*/*3.2*) and four of the genes in lemma (*TaBZR2.2*/*2.4*/*2.6*/*3.2*) showed the highest expression levels. In different development stages, some of the *TaBZR* genes showed significant expression. For example, the *TaBZR2.2*/*2.4*/*2.6* genes were gradually increased along with the root development. Moreover, among the 20 *BZR* genes, *TaBZR2.1*/*2.3*/*4.2*/*4.4*/*4.5* were barely expressed in all of the detected samples, which may have been due to them having spatial or temporal expression patterns that may not have been examined in our study. Overall, the expression profiles of the *TaBZRs* varied considerably. The differential expression patterns of the *TaBZR* genes could provide crucial clues for further investigating their biological function, implying that these genes may play different roles in wheat.

**Figure 8:**
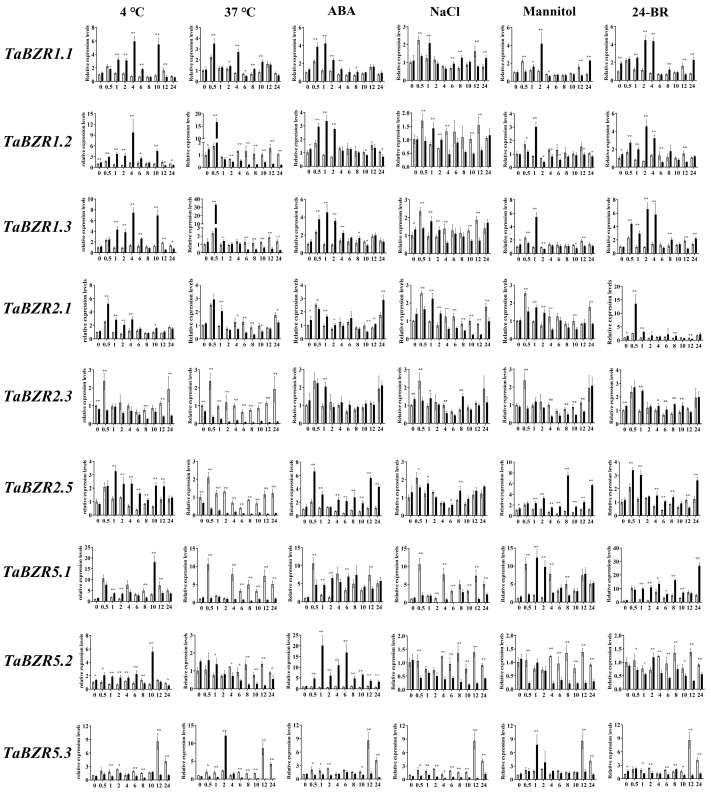
Expression analysis of the *TaBZR* genes in response to different treatments by qRT-PCR. Seeds of wheat cultivar Zhengmai366 (ZM366) were germinated for 3 days in the dark and then transferred to Hoagland liquid solution. The nutrient solution was changed every three days. At the trefoil stage (about three weeks old), seedlings were transferred to Hoagland liquid nutrient solution with ABA (200 mM), NaCl (200 mM), 20% PEG, and epi-BR (1μM) for ABA, NaCl, PEG, and epi-BR treatment, and seedlings were transferred to chambers at 37 °C or 4 °C to initiate heat and cold stress. The data were normalized with *TaACTIN* and *TaGAPDH*. The white and black columns in the diagrams represent the control and treatment groups, respectively. *, *p* < 0.05. **, *p* < 0.01.

Certain *BZR* genes were randomly selected from 20 *TaBZR* genes to further investigate the expression of *TaBZR* genes under various different treatments. A qRT-PCR assay was performed to analyze the expression levels under different treatments (Figure 8). Many of the *TaBZR* genes were markedly induced under various treatments. For example, under cold stress, the expression levels of *TaBZR* increased, and those of *TaBZR1.1*/*1.2*/*1.3* and *TaBZR2.1*/*2.3* initially increased and then later decreased. In the presence of heat and ABA treatments, the *TaBZR1.1*/*1.2*/*1.3* expression levels exhibited an intensive increase then decrease, while *TaBZR2.1*/*2.3* and *TaBZR5.1*/*5.3* were reduced. *TaBZR1.1*/*1.2*/*1.3* and *TaBZR2.1*/*2.2*/*2.3* were reduced under NaCl and mannitol treatments first, but they then increased, peaking after 1 or 2 h of treatment. In contrast, *TaBZR6* showed a continuous downregulation under cold, NaCl, ABA, mannitol, and 24-Epibrassinolide treatments. The expression levels of *TaBZR1.1*/*1.2*/*1.3* and *TaBZR2.1*/*2.2*/*2.3* were markedly increased under 24-BR treatments, and they peaked after 2 h or 0.5 h of treatment, implying that they play a crucial role in the BR signal pathway.

### 2.8. Subcellular Localization Analysis of TaBZR2.1 and Its Tissue-Specific Expression Analysis in Wheat

**Figure 9:**
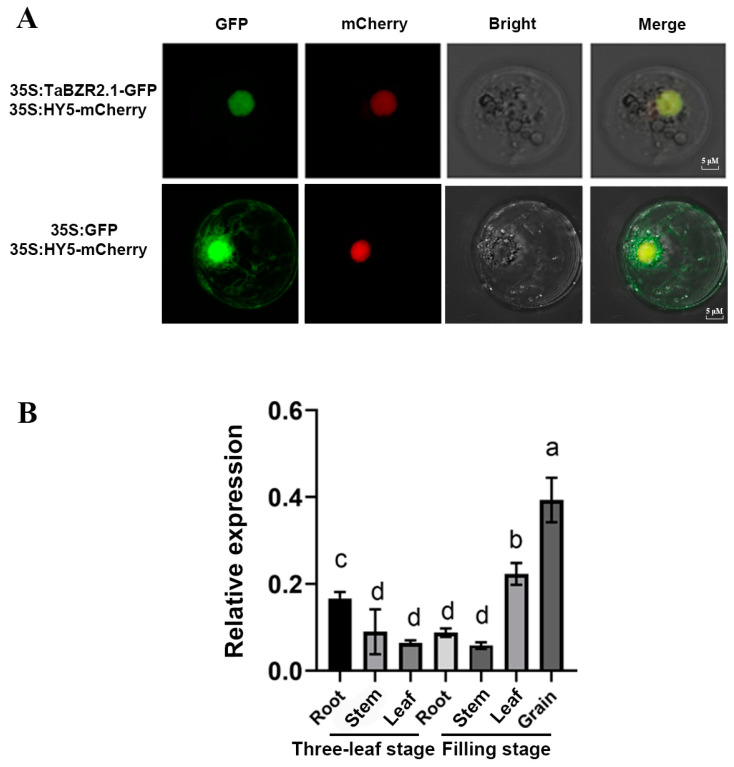
Subcellular localization of the TaBZR2.1-GFP fusion protein in the protoplast and TaBZR2.1 tissue-specific expressions. (**A**). TaBZR2.1-GFP protein driven by the 35S promoter were transiently expressed in protoplast cells of wheat, and they were observed using a confocal microscope. The GFP signals are represented by a green color; the red color represents the mCherry signals. Scale bars = 5 μM. (**B**). TaBZR2.1 tissue-specific expression profiles. Samples of the three-leaf and filling stages were collected, respectively, and the transcription levels of *TaBZR2.1* were measured using RT-qPCR assays, which were normalized with *TaACTIN* and *TaGAPDH*. The letters indicated significant at *p* < 0.05. Data are the mean ± SD (n = 3).

Since the *TaBZR2.1* gene responded to almost all of the treatments in the expression profile analysis of wheat *BZRs*, and the expression levels of *TaBZR2.1* initially increased in the early treatment stage and then later decreased, we chose *TaBZR2.1* for further analysis. The TaBZR2.1-GFP fusion vector, which was driven by the CAMV35S, was transformed into wheat protoplasts and expressed for 24 h to determine the subcellular localization of TaBZR2.1. The fluorescence signals were detected using confocal microscopy. The pCAMBIA2300-35S-EGFP vector and the pCAMBIA1300:35S:AtHY5-mCherry were used as the negative control and as a nuclear marker, respectively [39]. As shown in Figure 9A, the CaMV35S:GFP signal was observed in the whole cell, whereas the signal of TaBZR2.1-GFP was overlapped with 35S:AtHY5-mCherry. This result implies that the TaBZR2.1 protein was confirmed as a nuclear protein. In addition, the *TaBZR2.1* tissue-specific expression profiles showed that *TaBZR2.1* gene had a high expression in grain (Figure 9B).

### 2.9. Overexpression of TaBZR2.1 in Arabidopsis Decreases Brassinazole Resistance

**Figure 10:**
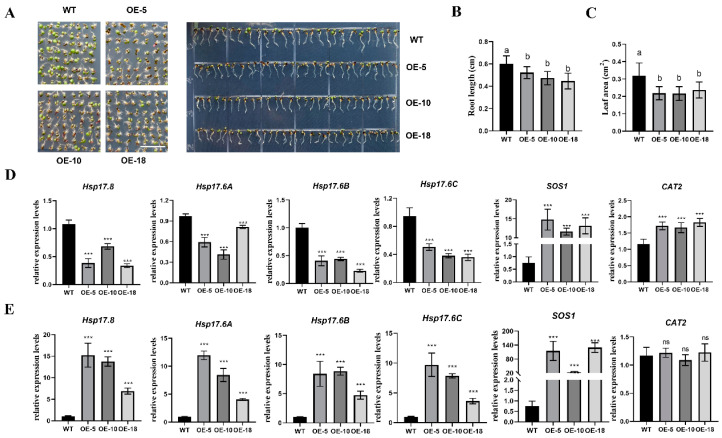
Overexpression of the *TaBZR2.1* gene in *Arabidopsis* negatively regulated the brassinazole-induced stress tolerance. (**A**). Seedling photographs of the 6-day-old Col-0 and TaBZR2.1-overexpressing *Arabidopsis* grown on 1/2 MS with brassinazole (1 μM). (**B**,**C**). The root length and leaf area of the seedlings in (**A**). The letters indicated significance at *p* < 0.05 (n = 30). (**D**,**E**). The expression levels of *Hsp17.8*, *Hsp17.6A*, *Hsp17.B*, *Hsp17.C*, *SOS1*, and *CAT2* in the seedlings under control (**D**) and brassinazole (**E**) treatment. ***, *p* < 0.001. ns, not significant.

The full-length *TaBZR2.1* was transferred into the *Arabidopsis* wild-type Col-0 to further investigate the molecular function of *TaBZR2.1*. A total of 20 T_1_ transgenic plants were obtained, and we selected three T_3_ homozygous lines for further investigation (Figure S1). Transgenic *Arabidopsis* lines were exposed to different treatments, including ABA, NaCl, mannitol, 24-Epibrassinolide, and brassinazole, to further investigate the role of the *TaBZR2.1* gene in stresses. As shown in Figure S2, on 1/2 MS medium, the overexpression lines showed no significant difference compared to WT. Meanwhile, on 1/2 MS medium with 1 μM of brassinazole, the overexpression lines exhibited a shorter root length and a smaller leaf area; that is, the transgenic lines were more sensitive to the brassinazole treatments compared to WT. Additionally, the expression of some of the stress-induced genes (including *AtHsp17.6A*, *AtHsp17.6B*, *AtHsp17.6C*, *AtHsp17.8*, *SOS1*, and *CAT2*) were quantified using RT-qPCR. The results showed that four of the small Hsps were downregulated in seedlings of the control group, while they were upregulated in the brassinazole treatment groups; the expression of *SOS1* and *CAT2* was upregulated both in the control and brassinazole treatments (Figure 10). Overall, the overexpressed *TaBZR2.1* plants displayed a greater sensitivity to brassinazole tolerance than the Col-0 plants, suggesting that the ectopic expression of *TaBZR2.1* brassinazole treatment negatively affects the root length and leaf area in *Arabidopsis*. It was also found that the *TaBZR2.1* overexpression lines were more sensitive to brassinazole-induced stress.

### 2.10. TaBZR2.1 Physically Interacts with Wheat AGO4

**Figure 11:**
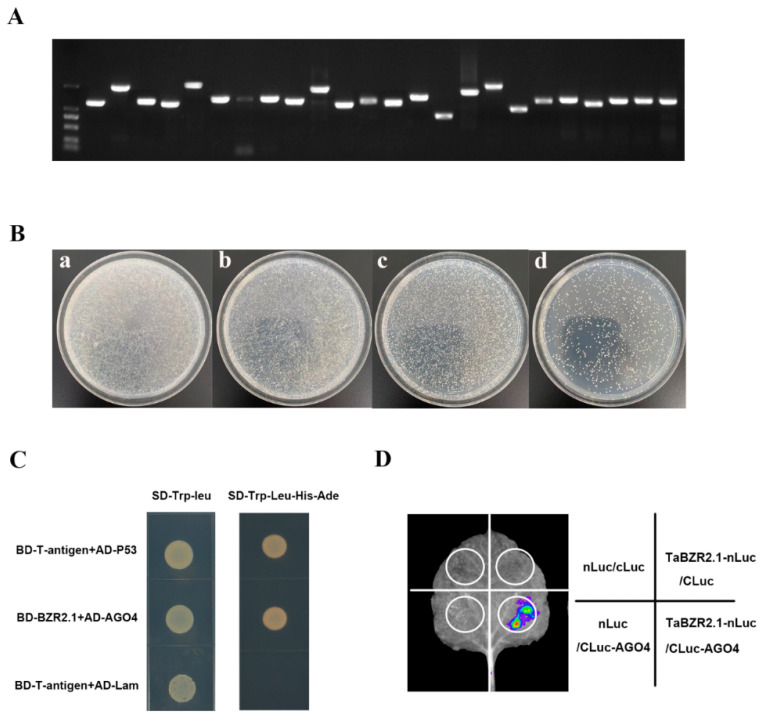
TaBZR2.1 physically interacting with AGO4. (**A**). Gel electrophoresis results for the identification of the insert fragments from the yeast library. (**B**). Yeast library titration. In the experiment, 100 μL of the 1/10, 1/100, 1/1000, and 1/10,000 dilutions and 100 μL of the yeast library were plated on SD/-Trp medium. Dilution factor = 10^−1^ (**a**), 10^−2^ (**b**), 10^−3^ (**c**), and 10^−4^ (**d**). (**C**). Yeast two-hybrid assay of TaBZR2.1 interacting with AGO4. BD-TaBZR2.1 (bait) and AD-AGO4 (prey) plasmids were transformed into the yeast (Y2H-gold) competent cell, as indicated and grown on the selection medium. (**D**). Split-LUC assay of TaBZR2.1 interacting with AGO4 in the tobacco leaves.

A Y2H library was constructed, and the quality of the library was qualified (Figure 11A,B). A Y2H screening was performed to elucidate the molecular mechanism by which TaBZR2.1 modulates, and a large number of proteins were enriched (Table S2). We were particularly interested in one protein, AGO4. The full-length CDS of AGO4 was cloned and it interacted with TaBZR2.1 in yeast (Figure 11C) Then, a split-LUC assay was performed to further confirm the interaction. We generated constructs encoding the nLuc and TaBZR2.1 fusion protein (TaBZR2.1-nLuc) and the cLuc and AGO fusion protein (cLuc-AGO4). Then, these constructs were infiltrated into tobacco leaves. Likewise, in the split-LUC assay, only constructs harboring TaBZR2.1 and AGO4 fused to the two halves of the luciferase protein showed strong LUC signals in the *N. benthamiana* leaves (Figure 11D), indicating TaBZR2.1 interacts with AGO4 in planta. Taken together, these results showed that TaBZR2.1 interacts with AGO4 in vivo and in vitro.

## 3. Discussion

### 3.1. Identification and Characteristic Analysis of Wheat BZR Genes

In this study, 20 *BZR* family genes in wheat were identified, which were named *TaBZR1.1* through *TaBZR6* on the basis of wheat gene symbolization guidelines (Figure 1). The genes in wheat usually have three homologs in three subgenomes (A, B, and D) because common wheat has endured two naturally interspecific hybridization events. However, in this study, no *BZR* gene was found on the 4A chromosome. The absence of the *BZR* gene on 4A could be the results of gene modification or the recombination events during the evolutionary process of the wheat [40,41]. Meanwhile, the synteny analyses showed that the *BZR* family expansion in wheat was generated by gene duplication events (Figure 4). In addition, three-dimensional modeling of TaBZRs was performed. Moreover, we found that the TaBZR proteins from the same subgenome showed similar models (Figure 3), and they were in agreement with previous findings of the phylogenetic and synteny analysis findings [42].

*BZR* genes can be induced by certain phytohormones and various stresses [43,44,45]. Previous study shows that maize *BZR* gene promoters contains light- and stress-responsive elements [33], while *Arabidopsis* and soybean *BZR* gene promoters revealed the presence of development-related and stress-, hormone-, and light-responsiveness-related cis-acting elements [46]. The cis-regulatory elements in the promoter region of *TaBZR* were analyzed (Figure 5). Certain *TaBZR* promoters included the GA-, ABA-, MeJA-, and SA-responsive elements, suggesting that some *TaBZR* genes might be regulated by phytohormone and stress conditions. It was observed in a previous study that some BZRs are key to regulating organ and seed sizes [5,23]. Moreover, some of the elements of seed-specific regulation and meristem expression elements have also been found in some *TaBZR* promoters. The results imply that the *TaBZR* genes might be involved in plant growth and development, the stress response, and phytohormone metabolism networks. Previous studies showed that the *BZR* genes can be induced by ABA, cold, drought, and salt stress [31]. And the expression of *ZmBZR* genes is responsive to ABA and light [33]. Similarly, in view of the crucial role played in the stress response, the expression levels of the *TaBZR* genes under cold (4 °C) and hot (37 °C) conditions, as well as under ABA, NaCl, mannitol, and 24-Epibrassinolide, were investigated in this study (Figure 8). The expression levels of most of the *TaBZR* genes dramatically changed when subjected to stress treatments.

Subcellular localization analysis is a valuable tool for gaining insights into the cellular activities of proteins [42], and the nuclear localization of proteins is a common feature of transcription factors [23]. Subcellular localization results showed that the TaBZR2.1 protein was localized in the nucleus (Figure 9A).

### 3.2. Molecular Functional Analysis of TaBZR2.1

*BZR* genes have been comprehensively investigated in rice and *Arabidopsis*, and they have been implicated in numerous plant processes. There is substantial evidence indicating that *BZR* genes play crucial roles in the regulation of plant growth, development, and response to environmental stress [30,31,34]. Recent studies have demonstrated that wheat *TaBZR2* provides resistance to wheat stripe rust [47]. Overexpression of *TaBZR1* in wheat increases grain size. TaBZR1 mediates wheat chilling tolerance by directly binding to the *TaSAMT1* promoter to activate its expression [48].

*BZR* genes respond to abiotic stress and plant hormone treatment. For instance, the overexpression of the tomato *BZR1D* gene in *Arabidopsis* positively regulates BR and salt tolerance [36]. By promoting ABA biosynthesis and ROS scavenging, the *TaBZR1* gene can enhance wheat salt tolerance. To dissect the molecular function of *TaBZR2.1*, *Arabidopsis* overexpression lines were generated using Agrobacterium-mediated transformation (Figure S1). We identified that the wheat *TaBZR2.1* gene is involved in the brassinazole stress response. In *Arabidopsis*, the ectopic expression of *TaBZR2.1* indicated the shoot root length and small leaf area under brassinazole treatment, and the *TaBZR2.1* overexpression lines were found to be more sensitive to brassinazole-induced stress (Figure 10). Overall, *TaBZR2.1* played a negative role in brassinazole stress response.

AGO proteins are present in various organisms and exhibit expression across a diverse array of tissues [49,50]. They are central components of the RNA-induced silencing complex (RISC) and play an important role in plant development and growth [51]. For example, *Arabidopsis* AGO1 regulates leaf and floral stem cell development by binding to miR172 and miR165/166 [3]. In rice, OsAGO17 regulates grain weight and seed size through miR172 [47]. Additionally, OsAGO18 can bind to miR168, thus participating in antiviral rice defense pathways [52]. In *Arabidopsis* and rice, the AGO1 gene is required for normal plant development [53]. In this study, the Y2H assay was used to identify TaBZR2.1-interacting proteins. Our findings indicated that TaBZR2.1 directly interacts with AGO4, as predicted, suggesting that BZR-mediated BR signaling may serve as an important strategy in miRNA-mediated gene regulation (Figure 11C,D).

Overall, our findings provide valuable clues about the *TaBZR* family genes and their functional response to a variety of hormones and stresses in the different plant developmental processes. However, the molecular functions of *TaBZR* genes need to be further investigated.

## 4. Materials and Methods

### 4.1. Identification of BZRs in Wheat

The sequences of DNA (FASTA), proteins (FASTA), and the GFF3 files of wheat were downloaded from EnsemblPlants. The hidden Markov model of the BES1/BZR plant transcription factor N-terminal (Pfam login number: IPR008540) was obtained from the Pfam database and BLAST tool was used to identify the homologous proteins in wheat, maize, rice, and *Arabdopsis*. The obtained protein sequences were further analyzed by the NCBI Conserved Domain Database, and the redundant sequences were removed. The 20 *BZR* genes were named in accordance with gene symbolization guidelines.

### 4.2. Chromosomal Localization and Collinearity of the TaBZR Genes

The *TaBZR*s were mapped onto the chromosomes by analyzing the wheat GFF3 files. We utilized the TBtools software (V1.098, Guangzhou, China) [54] and MCScanX toolkit program (Athens, OH, USA) to visualize the chromosomal localizations and gene duplications.

### 4.3. Phylogenetic Analysis of BZR Proteins

The protein sequences of the BZR family in wheat and other selected species were downloaded and aligned via MUSCLE [55] using the default parameters to assess the evolutionary relationships of *BZR* in plants. Using MEGA-X software (V11.0., Philadelphia, PA, USA), a neighbor-joining phylogenetic tree was generated. Evolview software (V4.0, Beijing, China) was used to further modify the tree.

### 4.4. Subcellular Localization of TaBZR in Wheat Protoplasts

The coding sequence (CDS) of *TaBZR2.1* (gene ID: TraesCS3A02G123500) was PCR-amplified from the cDNA of the Zhengmai 366 genetic background of wheat using specific primers and KOD OneTM PCR Master Mix (TOYOBO, KMM-101, Osaka, Japan), and it was cloned into the pAN580-GFP vector. The HY5 protein of *Arabidopsis* was used in this study as a nuclear marker [27]. These vectors were then transformed into wheat protoplasts. The steps of the wheat protoplast extraction and transformation were conducted as has been described in a previous study [56]. The GFP fluorescence signals of the protoplast were observed under a confocal microscope (Zeiss, Jena, Germany).

### 4.5. Plant Materials and Stress Treatments

Healthy seeds of wheat cultivar Zhengmai366 (ZM366) were germinated in distilled water for 3 days in the dark. The germinated seeds were then transferred into a Hoagland liquid nutrient solution. At the trefoil stage, the seedlings were transferred to nutrient solution with ABA (final concentration 200 mM), NaCl (final concentration 200 mM), 20% PEG, and epi-BR (final concentration 1μM) for the ABA, NaCl, PEG, and epi-BR treatments, respectively. Meanwhile, trefoil-stage wheat was transferred into chambers at either 37 °C or 4 °C to initiate heat and cold stress. The solution was changed every three days. Leaf samples were collected at different time points after treatment.

The full-length open reading frame (ORF) of the *TaBZR2.1* gene was cloned into the Kpn I/Sal I-digested 35S-eGFP vector to generate transgenic plants overexpressing *TaBZR2.1*. These constructs were then transformed into *Arabdopsis* (Col-0) via the *Agrobacterium* dipping flower method. The genomic DNA and RNA of the T1 plant were extracted to verify the presence of overexpression genes, and the expression levels of the *TaBZR2.1* gene and the specific primer pairs were used for PCR amplification (Table S3). Seeds of the T1 transgenic lines were sterilized and sown on a 1/2 MS medium containing kanamycin (final concentration 50 μg/mL) for further investigation.

### 4.6. Gene Expression Pattern Analysis

The RNA of samples collected in this study was extracted using an RNAiso Plus (No: 9108, Takara, Osaka, Japan). The quantity and quality of the extracted RNA from samples were examined using a Qubit 4.0 Fluorometer (Invitrogen, Carlsbad, CA, USA). The RNA were then reverse-transcribed to cDNA for real-time qPCR analysis. The expression levels of *TaBZR2.1* genes were normalized against those of *TaACTIN* (gene ID: TraesCS5B02G124100) and *TaGAPDH* (gene ID: TraesCS6B02G243700). The transcriptomic data from different tissues were downloaded from the Wheat eFP Browser. A hierarchical cluster map was obtained by the Tbtools software (V1.098, Guangzhou, China) [54].

### 4.7. Yeast Two-Hybrid Assay

A Y2H experiment was performed in accordance with the Matchmaker Gold Yeast Two-Hybrid System (https://www.takarabiomed.com.cn/, accessed on 20 November 2024). To generate the pGBKT7-TaBZR2.1 bait vector, the CDS of *TaBZR2.1* was amplified from the cDNA of wheat and cloned into the digested pGBKT7 vector. The CDS of *AGO4* (gene ID: TraesCS3A02G188400) was amplified and ligated into the pGADT7 plasmid in order to generate the prey vector, producing pGADT7-AGO4. Then, the prey vectors were co-transformed with bait vectors. The transformants were plated on DDO (SD/-Leu/-Trp) and QDO medium (SD/-Ade/-His/-Leu/-Trp) with ABA (125 ng/mL).

### 4.8. Split Luciferase Complementation Assay

cLuc-AGO4 and TaBZR2.1-nLuc vectors were used in the analysis. Constructs were electroporated into the agrobacterium competent cell (GV3101) and then injected into *N. benthamiana* leaves. After 48 h of infiltration, tobacco leaves were sprayed with D-luciferin (final concentration 1 mM), and then removed from light for 5 min. The signals were detected using a Night SHADE LB 985 system (Berthold, Stuttgart, Germany).

### 4.9. Conserved Protein Motifs and Structure of the TaBZRs

We used the MEME software (V5.5.7, Reno, NV, USA) to analyze the conservative motifs of the TaBZRs, and the results were visualized using the TBtools software (V1.098, Guangzhou, China) [54]. The exon–intron structures of the *TaBZR* genes were analyzed using GSDS 2.0.

### 4.10. Statistical Analysis

The IBM SPSS Statistics 22 software (Chicago, IL, USA) was used in this study to analyze the data using one-way ANOVA.

## 5. Conclusions

A total of 20 *TaBZR* gene members were identified in wheat in this study, and comprehensive bioinformatic analyses were performed for them. The promoter regions of the *TaBZR* genes contained elements associated with stress responses and the phytohormone response. Meanwhile, we confirmed that the TaBZR2.1 protein was located in the nucleus. In addition, we observed TaBZR2.1 physically interacting with AGO4 in vivo and in vitro. The discoveries in this study offer valuable clues for further investigation of *BZR* genes in wheat.

## Data Availability

No new data were created.

## Supplementary Materials

The following supporting information can be downloaded at https://www.mdpi.com/article/10.3390/ijms252312545/s1.
