# Supplementary material for: Identification of the Brassinazole-Resistant (BZR) Gene Family in Wheat (Triticum aestivum L.) and the Molecular Cloning and Functional Characterization of TaBZR2.1
Source: Int J Mol Sci. 2024 Nov 22;25(23):12545. doi: 10.3390/ijms252312545 (PMC11641346; doi:10.3390/ijms252312545)
Supplement: Supplementary file 1 [file ijms-25-12545-s001.zip › Supplementary File/Supplemental Figures.doc]

**Supplementary Figures**

**Figure S1.** Identification of the *TaBZR2.1* transgenic *Arabidopsis* lines.

A. Diagram of the pCAMBIA2300- TaBZR2.1-eGFP vector.

B. PCR analysis of *TaBZR2.1* in 20 *Arabidopsis* transgenic lines. “+” and “-” represented positive and negative control, respectively. The number 1－20 represents *TaBZR2.1* transgenic *Arabidopsis* lines.

C. Relative expression levels of *TaBZR2.1* genes in 20 T3 *Arabidopsis* transgenic lines, and the data were normalized with the *ACTIN 2* (AT3G18780) gene.

Figure S2 Phynotypes of TaBZR2.1 over-expression *Arabidopsis* in on 1/2 MS.

A. Phynotypes of 6-day-old wild-type and of TaBZR2.1-overexpressed *Arabidopsis* seedlings grown on ^1^/_2_ MS.

B,C. The leaf area and root length of seedlings in A, N ≥ 25.
